# Autonomic and peripheral neuropathy with reduced intraepidermal nerve fiber density can be observed in patients with gastrointestinal dysmotility

**DOI:** 10.1002/ccr3.2575

**Published:** 2019-12-04

**Authors:** Bodil Ohlsson, Lars B. Dahlin, Elisabet Englund, Béla Veress

**Affiliations:** ^1^ Department of Internal Medicine Skane University Hospital Lund University Malmö Sweden; ^2^ Department of Translational Medicine – Hand Surgery Lund University Malmö Sweden; ^3^ Department of Hand Surgery Skåne University Hospital Malmö Sweden; ^4^ Department of Pathology Skåne University Hospital Lund Sweden; ^5^ Department of Pathology Skåne University Hospital Malmö Sweden

**Keywords:** autonomic dysfunction, enteric neuropathy, gastrointestinal dysmotility, intraepidermal nerve fiber density, peripheral neuropathy

## Abstract

Neuropathy should be considered as a possible etiological factor in patients with severe gastrointestinal symptoms, without signs of disease on routine investigations. Examinations of the autonomic and peripheral nervous systems may be helpful to select the patients who should be investigated with full‐thickness intestinal biopsy, and to give appropriate care.

## Graphical abstract

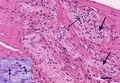

## 1 INTRODUCTION

Severe gastrointestinal (GI) dysmotility in the form of chronic intestinal pseudo‐obstruction (CIPO) and enteric dysmotility (ED) are rare diseases, which are difficult to diagnose.^1^ Enteric neuropathy, leading to dysmotility, is seldom studied, since there are few centers available for evaluation of GI motility. Furthermore, there are difficulties to get access to myenteric plexus of the enteric nervous system, and the histopathological examination of tissue samples is time‐consuming. Subsequently, most patients with GI dysmotility and pain are diagnosed to have irritable bowel syndrome (IBS), or the neuropathy diagnosis is delayed for several years.^2^, ^3^ Due to different treatment strategies between GI dysmotility and IBS, it is important to diagnose the diseases correctly. Functional bowel diseases are mainly treated with dietary advice in the form of regular meals with carbohydrate restriction and psychological therapy,^4^ whereas dysmotility is treated with prokinetic drugs and dietary supplemental therapy.^5^

Previous reports have shown signs of autonomic neuropathy and serum antibodies against neural components in patients with CIPO and ED.^3^, ^6^ Furthermore, autonomic and peripheral neuropathy are associated, for example, in diabetes mellitus.^7^ This has led to the hypothesis that examination of the autonomic and peripheral nervous systems could be used, to select candidates for full‐thickness intestinal biopsy, and to facilitate the differentiation between patients with neuropathic dysmotility and IBS.

The present double case report describes two women with severe GI dysmotility during several years, confirmed by examinations of GI motility and full‐thickness biopsy with immunohistochemical staining. In 2018, examinations with specific questionnaires regarding symptoms of autonomic and peripheral dysfunction, along with autonomic nerve function tests and skin biopsies, were performed to examine the autonomic and peripheral nervous systems.

Patients with gastrointestinal dysmotility may have symptoms and signs of a more generalized neuropathy. Besides examining the gut function, also the autonomic and peripheral nerve functions with determination of the intraepidermal nerve fiber density should be tested, to select patients for full‐thickness intestinal biopsies and to handle the patients appropriately.

## 2 MATERIALS & METHODS

This study was performed according to the Declaration of Helsinki and approved by the Regional Ethics Review Board at Lund University (approval number 2016/217, 2018/132). The patients gave their written informed consent for participation in the study and publication of the results.

### 2.1 Questionnaires

A questionnaire concerning the symptoms usually present in patients with gastric paresis and/or GI dysmotility,^8^ the validated Autonomic Symptom Score,^9^ and the validated Neuropathy Symptom Score^10^, ^11^ were completed by the patients.

### 2.2 CIPO analyses

Full‐thickness biopsies of the small intestinal wall, 1.5 × 1.0 cm, were obtained through surgery in full anesthesia, according to a protocol for CIPO analysis,^3^, ^12^ which includes immunostaining of neural tissue and smooth muscle cells for disease classification.^13^, ^14^ Replacement of specific antibodies by nonimmunized serum was used as negative controls. Stained sections of normal parts from four small bowel resections due to carcinoma were used as positive controls.

### 2.3 Quantitative analysis of various cell types

All neurons with nuclei and large perikaryons, with or without protein gene product (PGP 9.5)/bcl‐2 immunoreactivity, were counted, and the percentage of negative cells was determined. Furthermore, all CD117‐positive, nucleated interstitial cells of Cajal (ICCs) were recorded within the myenteric plexus and in the circular muscle layer, including the deep muscular plexus. The CD3‐positive T‐lymphocytes were counted as previously described.^12^

### 2.4 Autonomic nerve function tests

Deep breathing test,^15^ orthostatic tilt table test,^16^ and laser Doppler perfusion imaging^17^ were performed according to clinical routines.

### 2.5 Skin biopsies and immunohistochemistry

Skin biopsies and histopathologic immunohistochemistry were performed according to clinical routines as previously described for the assessment of intraepidermal nerve fiber density (IENFD), that is, number of complete nerves/mm.^18^, ^19^ In brief, a 3‐mm punch biopsy, taken during local anesthesia about 10 cm proximal to the lateral malleolus in the lower leg, was fixed in formaldehyde solution, embedded in paraffin and sectioned at 4 µm, and then stained with a polyclonal antibody against PGP 9.5.^18^ Distinct from previous accounts, the antibody was of a new brand (Cellmarque/318A‐15; Sigma Aldrich^©^; dilution 1:100). Two women, 31 and 58 years old, served as healthy controls for comparison of the IENFD.

## 3 CASE EHLERS‐DANLOS SYNDROME

### 3.1 Enteric neuropathy

A 69‐year‐old woman suffered from abdominal pain, often called irritable bowel syndrome (IBS), since childhood. Already at the age of 5‐6 years, she had an attack of subileus. Since the 1980s, the GI symptoms have accelerated. Reflux was a predominant symptom, and antireflux surgery was performed twice during this decade. In parallel, she got the diagnosis Ehlers‐Danlos syndrome, in accordance to the present criteria.^20^ In addition, she also suffered from autoimmune thyroiditis.

**Figure 1:**
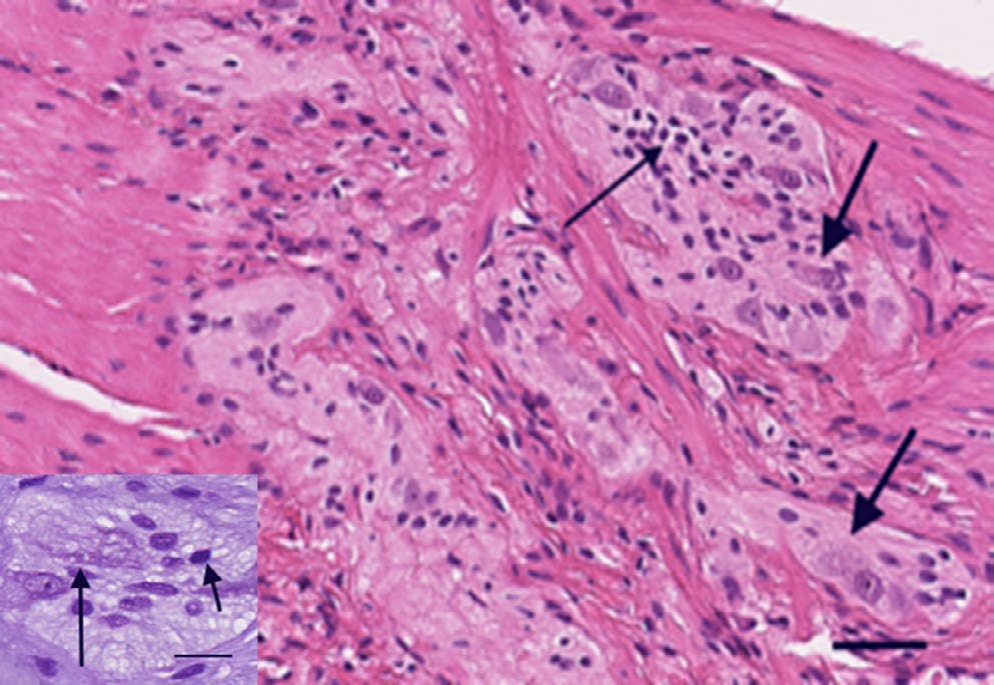
Horizontally cut section with myenteric ganglia. Within the uppermost ganglion one group of lymphocytes are seen (long arrow). Thick arrows show two degenerating neurons with vacuolated “bunch of grape‐like” cytoplasm. Inset: Myenteric neuron with vacuolated cytoplasm and chromatolysis. Nissl‐material is marginalized under the plasma membrane (long arrow). Short arrow indicates a lymphocyte. Hematoxylin & eosin, bar: 25 µm; inset: cresyl violet, bar: 10 µm

Gastric scintigraphy and transit time examination showed prolonged gastric emptying (16 h), and antroduodenal manometry showed abnormal contractile activity with abnormal bursts. Full‐thickness biopsy of the bowel wall was performed in 2006. The small intestinal mucosa, submucosa, submucosal neurons, and ganglia showed no histopathological changes. In the myenteric plexus, several of the ganglia were small and contained few neurons, with or without intraganglional lymphocytes, whereas other ganglia were of normal size without lymphocytes (Table 1). Several neurons with vacuolization were enlarged and swollen. Some of the vacuolated neurons were similar to “bunch of grapes” (Figure 1). Diastase resistant periodic acid‐Schiff (PAS)‐positive lipofuscin and coarse vimentin‐positive granules were found in many neurons. Eleven percent of the neurons were negative for bcl‐2 (control: 100% positivity). There were peri‐ and intraganglional T cells with one larger focus of cells (Figure 1; Table 1). T cells were observed partly within the interganglional nerve bundles of the myenteric plexus, and partly alongside the small intramuscular nerves. Hyperplasia and hypertrophy of the ICCs were detected both in the myenteric ICC plexus and in the circular muscle layer (Table 1). Around the small ganglia with diminished number of neurons, no ICC projections could be seen in the immunostained sections. The longitudinal muscle layer was thickened with hypertrophic myocytes (circular/longitudinal layers: 0.65/0.6 mm; quotient: 1:1; normal quotient: 1.5‐2.0). The histopathological diagnosis was myenteric lymphocytic ganglioneuritis with vacuolar neurodegeneration and hyperplasia of the ICC and secondary hypertrophy of the longitudinal muscle layer.

**Table 1:** Histopathological findings in small intestine and skin

Organ	Types of cells	Ehlers‐Danlos syndrome (mean)	Drug‐induced dysmotility (mean)	Controls (mean)
Small bowel	Myenteric neurons PGP9.5+	58 neurons/10 mm	59 neurons/10 mm	125 neurons/10 mm Range: 65‐154
	ICC‐CM	33 ICC/mm^2^	48 ICC/mm^2^	Upper normal value: 14 ICC/mm^2^
	ICC‐MP^a^	54 ICC/mm	42 ICC/mm	Upper normal value: 24 ICC/mm
	T‐lymphocytes^b^	0.7 cells/ganglion Range: 0‐25	0.35 cells/ganglion Range: 0‐2	0.17 cells/ganglion Range: 0‐4
Skin	Intraepidermal nerves	0 nerve/mm	0.5 nerve/mm	5 nerves/mm

Abbreviations: +, positive; CM, circular muscle; MP, myenteric plexus.

a Interstitial cells of Cajal (ICC)‐number per mm intermyenteric connective tissue plate.

b Mean number of peri/intraganglional T cells/ganglion; normally no T cells within ganglion or interganglional nerves, <5 periganglional T cells normal.^12^

In 2007, she had volvulus, which resulted in hemicolectomy. During the last 20 years, she has had marked difficulties to tolerate carbohydrates and has had postprandial hypoglycemia, which has compelled her to eat a carbohydrate‐restricted diet. Analysis of autoantibodies in serum did not show any presence of gonadotropin‐releasing hormone (GnRH) antibodies. She full‐filled the vast majority of symptoms of gastric paresis (Table 2), as well as symptoms of both diarrhea and constipation, and the clinical diagnosis of ED was set.^1^ She was treated with proton pump inhibitors (PPI), analgesic drugs, antidepressant drugs, laxatives, and antidiarrheal drugs to improve symptoms. The disease has progressed slowly with more symptoms over time.

**Table 2:** Symptoms of gastrointestinal dysmotility and autonomic neuropathy

Symptoms of gastroparesis/gastrointestinal dysmotility	Ehlers‐Danlos syndrome	Drug‐induced dysmotility
Loss of appetite	+	+
Dysphagia	+	+
Meal‐related cough	+	+
Early satiety	+	+
Nausea	+	+
Vomiting	0	+
Weight loss	+	0
Abdominal fullness	+	+
Bloating	+	+
Regurgitation	+	+
Constipation	+	+
Diarrhea with gas	+	+
Evacuation incontinence	+	0
Postprandial glycemia pitfalls	+	+
Symptomatic postprandial hypoglycemia	+	0
Postprandial perspiration	+	0
Symptoms of autonomic neuropathy		
Postural hypotension	2	2
Sphincter loss	1	1
Nocturnal diarrhea	1	1
Postprandial perspiration	2	0
Symptoms of gastric atony	2	2
Absence of symptoms from hypoglycemia	0	1
Impotence	0	0

Note

0 = never, 1 = sometimes, 2 = often, + = presence of symptoms without determination of degree of symptoms. Questionnaires of gastric paresis/gastrointestinal dysmotility^8^ and autonomic symptom score.^9^

### 3.2 Autonomic neuropathy

The most prominent symptoms were postural hypotension, postprandial perspiration, and symptoms of gastric atony (Table 2). A tilt table test showed hypotension, and the diagnosis postural orthostatic tachycardia syndrome (POTS) was set.^21^ The blood flow in the skin circulation was generally low.

### 3.3 Peripheral neuropathy

She was diagnosed in 1995 with polyneuropathy in legs and feet. She was treated with right‐sided neurolysis due to a carpal tunnel syndrome in 2009 and a cubital tunnel syndrome in 2011 and experienced also paresthesia and numbness on the left side. She had frequent symptoms of peripheral neuropathy from both hands and feet, also during the nights (Table 3). Histopathological examination revealed reduced IENFD, with one single nerve fiber, shorter than half of the epidermal thickness. In contrast, the controls showed 10 long nerves/biopsy, that is, 5 nerves/mm (Table 1).

**Table 3:** Symptoms of peripheral neuropathy

Symptoms of peripheral neuropathy	Ehlers‐Danlos syndrome		Drug‐induced dysmotility	
	Feet	Hands	Feet	Hands
Numbness	2, 3	3	2	2
Cold‐heat feeling	3	0	2	2
Paresthesia	2, 3	2, 3	1	1
Burning pain	2, 3	0	1	1
Radiation pain	2, 3	2, 3	2	2
Constant pain	2, 3	0	2	2
Irritations by sheets in bed	3	0	1	1
Symptom duration (years)	17	11	2	3

Note

0 = never, 1 = sometimes, 2 = often, 3 = nocturnal. The neuropathy symptom score.^10^, ^11^

## 4 CASE DRUG‐INDUCED DYSMOTILITY

### 4.1 Enteric neuropathy

A 49‐year‐old woman acquired acute chest and abdominal pain combined with dysphagia at the age of 30 years, in relation to her 4th in vitro* fertilization* (IVF) with the GnRH analog buserelin. The symptoms progressed along with abdominal pain, vomiting, constipation, bloating, and extensive belching. A thorough examination revealed a swallowing defect, impaired esophageal motility lacking peristalsis, gastroesophageal reflux, and delayed gastric emptying rate (75% of the test meal remained in the stomach after 70 min; T50 in controls: 40 ± 28 min). Enterography demonstrated extremely slow passage through the stomach and small intestine with distention of the GI tract and antroduodenal manometry displayed abnormal fasting bursts. No organic abnormalities of thorax and upper abdomen could be find on computer tomography, and extensive analyses of liquor and blood revealed antibodies against GnRH in serum.

The detailed histopathological analysis of the enteric neurons with typical microscopic photographs has been published previously.^22^ Briefly, the mucosa, submucosa, and submucosal ganglia and neurons were normal. The total number of myenteric neurons was significantly decreased compared to controls (Table 1; *P* < .01), and 20% of the neurons of the myenteric plexus in the patient were either swollen, rounded with a smooth surface with or without vacuolization of the cytoplasm, or were shrunken with pyknotic nuclei and amphophilic cytoplasm. The immunoprofile was abnormal: 20% of the neurons were negative or very weakly stained for PGP 9.5 (control: 94% positivity) and 13% of the neurons were negative for bcl‐2 (control: 100% positivity). The small intramuscular nerves were markedly thickened with axon vacuolization. There were hyperplasia and hypertrophy of the ICCs, as well as cytoplasmic vacuolization, in both the myenteric plexus and circular muscle layer (Table 1). The longitudinal muscle layer as well as the internal circular muscle layer was thickened, with enlarged hypertrophic smooth muscle cells. The histopathological diagnosis was visceral degenerative neuropathy with axon vacuolization, hyperplasia, and vacuolization of ICCs, and hypertrophy of the longitudinal and internal circular muscle layers.

The patient's general condition deteriorated, with the most pronounced symptoms according to the patient being abdominal pain, bloating, and constipation, followed by periods of diarrhea. The clinical diagnosis of CIPO was set.^1^ She was treated with analgesic and prokinetic drugs, and laxatives during periods of severe constipation. In periods, a nasojejunal tube was used to support nutritional intake.

### 4.2 Autonomic neuropathy

The most pronounced symptoms of autonomic dysfunction were postural hypotension and symptoms of gastric atony (Table 2). Deep breathing test and repeated orthostatic tilt table tests were normal. An abnormal vasoconstriction (z‐score = 2.97 compared with reference value <1.64 [11]) was observed in 2003, indicating sympathetic neuropathy.

### 4.3 Peripheral neuropathy

The most pronounced symptoms of peripheral neuropathy were numbness, cold‐heat feeling, and pain in both hands and feet, appearing the last 2‐3 years (Table 3). However, electrophysiology examination did not show any disturbances in the median, ulnar, peroneal, or sural nerves. Histopathological examination of skin biopsy revealed a markedly reduced IENFD, with only small nerve fiber fragments, and only one single long nerve fiber within the epidermal layer (Table 1).

## 5 DISCUSSION

The present two cases represent patients with severe GI dysmotility secondary to Ehlers‐Danlos syndrome or drug treatment, who also have autonomic and peripheral neuropathy. Thus, the enteric neuropathy can be considered to be a part of a generalized neuropathy in the enteric, autonomic, and peripheral nervous systems.

Generalized neuropathy has previously been described in diabetes mellitus.^7^, ^23^ In accordance, diseases, such as amyloidosis and Fabry disease, characterized by amyloid deposits and progressive lysosomal accumulation of lipids, respectively, have been described to develop both severe GI dysmotility, as well as autonomic and peripheral neuropathy.^24^, ^25^ GI symptoms secondary to Ehlers‐Danlos syndrome are well‐known in clinical practice, often entitled as functional bowel disorders.^26^ However, severe GI dysmotility in the form of CIPO or ED in Ehlers‐Danlos syndrome has only been described in the literature as case reports of children.^27^, ^28^ Our patient with Ehlers‐Danlos syndrome is, to the best of our knowledge, the first report of an adult patient with severe GI dysmotility and histopathological changes in the enteric nervous system. Clinical examination revealed severe GI dysmotility called ED and aggravated histopathological findings of depletion of both enteric neurons and ICC. The dysmotility with a flaccid GI tract may be the reason to both reflux symptoms and volvulus. The symptom diarrhea and constipation varied over time. Some of the GI symptoms could possibly be ascribed to dumping secondary to antireflux surgery. However, dumping secondary to this surgery is very rare.^29^

The pathophysiology behind the GI dysmotility and generalized neuropathy in Ehlers‐Danlos syndrome is unknown. One hypothesis is lack of supportive tissue to the blood vessels, with ensuing hypoxia in the organs.^30^ The possible role of telocytes, which could be responsible among others for the homeostasis of the interstitium and blood vessels,^31^ could influence the blood supply of the myenteric ganglia leading to neurodegeneration. Furthermore, we do not know whether there is a connection between Ehlers‐Danlos syndrome and the lymphocytic myenteric ganglioneuritis, or both are primary, concomitant diseases in our patient.

GnRH analogs have been shown to induce enteric neurodegeneration in some patients. The mechanism is postulated to depend on an increased initial luteinizing hormone (LH) release with overstimulation of LH receptors in the bowel wall, leading to neuronal death.^32^ Patients with polymorphism of the LH receptor are more vulnerable to be damaged from GnRH analogs, than patients without this polymorphism.^32^ The effect of GnRH on other parts of the nervous system is not examined, but may be possible since GnRH receptors are widely spread in the brain and spinal cord.^33^ GnRH may act as a causal agent to disturbances in both the central nervous system and afferent and efferent pathways between the gut and brain, as well as in peripheral pathways, in similarity to disturbances developed in diabetes mellitus.^7^ Further, afferent signals from the GI tract are transformed through parasympathetic and sympathetic neurons and further dispersed to thalamus, hypothalamus, and several other brain centers. Consequently, these neuronal circuits suggest that the enteric nervous system, which is a part of the autonomic nervous system, may also affect central autonomic functions in a secondary way.^34^

When questionnaires regarding symptoms of autonomic and peripheral neuropathy were administered, several symptoms were present. This underlines the importance of a careful examination of the medical history, and stresses the importance to use specific questionnaires, to discover and put attention to symptoms from other organs than the GI tract. These symptoms had been overlooked by the clinicians until the questionnaires were completed, although a history of surgical treatment of carpal and cubital tunnel syndromes in the first case was present. One should consider the possibility of an increased susceptibility for nerve compressions disorders when a peripheral neuropathy is present, as described in diabetes mellitus.^35^, ^36^

The presence of symptoms and signs of autonomic and peripheral neuropathy, may improve the possibility to correctly and early diagnose a generalized neuropathy. Common imaging techniques such as computed tomography or endoscopy are not useful to study GI dysmotility. Rather, examinations of the function of motility and/or histopathological examinations are necessary to set a correct diagnosis. Both autonomic and neurophysiological tests, as well as skin biopsies, are cheaper than expensive dysmotility examinations, for example, antroduodenal manometry, and the accessibility to both examinations and tissue sampling is higher. Furthermore, the side effects, and inconvenience for the patients, are negligible compared to full‐thickness biopsy with the risk of bowel perforation and anesthetic complications. Thus, these examinations could be performed first, to be helpful to select the patients who should be considered for full‐thickness biopsy. The correct diagnosis of enteric neuropathy has had consequences for the clinical handling of the patients. GI surgery of rectocele, which had been performed if the patient had been diagnosed with IBS, was avoided. One of the patients has been treated with intermittent nasojejunal feeding, which is not a treatment option in IBS. Also, the drug treatment has been focused on the objective findings of GI dysmotility, with prokinetic drugs.

In conclusion, this report of two cases indicates that neuropathy may be a generalized disorder in patients with GI dysmotility, and not only restricted to the enteric nervous system. It further stresses the importance to examine the presence of symptoms or signs of autonomic and peripheral neuropathy, which also have to be appropriately treated. Examinations with low inconveniences and risks for the patients are to prefer as initial steps of the investigation. A diagnosis of neuropathy instead of IBS leads to a different therapeutic approach for the individual patient.

## CONFLICT OF INTEREST

There are no conflicts of interest.

## AUTHOR CONTRIBUTION

All authors designed and planned the study together. LD performed the skin biopsy. EE examined the histopathology of the skin biopsy. BV examined the histopathology of the intestinal biopsy. BO wrote the first draft of the manuscript. All authors contributed intellectually in the writing process and accepted the final version.

## Data Availability

Data can be obtained after request.
